# Radical polymerization by a supramolecular catalyst: cyclodextrin with a RAFT reagent

**DOI:** 10.3762/bjoc.12.244

**Published:** 2016-11-22

**Authors:** Kohei Koyanagi, Yoshinori Takashima, Takashi Nakamura, Hiroyasu Yamaguchi, Akira Harada

**Affiliations:** 1 Department of Macromolecular Science, Graduate School of Science, Osaka University, Toyonaka, Osaka 560-0043, Japan; 2 JST-ImPACT, Chiyoda-ku, Tokyo 100-8914, Japan

**Keywords:** cyclodextrin, radical polymerization, RAFT polymerization, substrate recognition site, supramolecular catalyst

## Abstract

Supramolecular catalysts have received a great deal of attention because they improve the selectivity and efficiency of reactions. Catalysts with host molecules exhibit specific reaction properties and recognize substrates via host–guest interactions. Here, we examined radical polymerization reactions with a chain transfer agent (CTA) that has α-cyclodextrin (α-CD) as a host molecule (α-CD-CTA). Prior to the polymerization of *N*,*N*-dimethylacrylamide (DMA), we investigated the complex formation of α-CD with DMA. Single X-ray analysis demonstrated that α-CD includes DMA inside its cavity. When DMA was polymerized in the presence of α-CD-CTA using 2,2'-azobis[2-(2-imidazolin-2-yl)propane dihydrochloride (VA-044) as an initiator in an aqueous solution, poly(DMA) was obtained in good yield and with narrow molecular weight distribution. In contrast, the polymerization of DMA without α-CD-CTA produced more widely distributed polymers. In the presence of 1,6-hexanediol (C_6_ diol) which works as a competitive molecule by being included in the α-CD cavity, the reaction yield was lower than that without C_6_ diol.

## Introduction

The folding of proteins in biological systems, the replication of DNA, and specific substrate recognition by enzymes play important roles in forming supramolecular structures, achieving functions, and maintaining life [1–6]. The crystal structures of RNA polymerase, DNA polymerase, and λ-exonuclease demonstrate that the cylindrical cavities of enzymes can effectively recognize substrates to produce biological polymers [1–6]. Cyclodextrins (CDs) have been widely used as substrate-recognition moieties in artificial enzymes [7–15], which have been used in the hydrolysis of activated esters [16–19] and as phase-transfer catalysts [20–28]. Moreover, via complex formation, modern supramolecular catalysts [29–33] have been used to achieve various highly efficient and selective reactions, including hydrolysis reactions [10–15], C–H bond activation [34–36], olefin epoxidation [37–39], Diels–Alder reactions [40–42], 1,3-dipole cycloadditions [43–44], and polymerizations [45–47], among others. Selective substrate recognition and activation are essential functions of supramolecular catalysts.

CD derivatives are widely used in radical polymerization to dissolve hydrophobic monomers in aqueous solutions [48–54] and to control the aggregation of polymers [55–58]. Although supramolecular catalysts with CDs as monomer recognition sites and catalytic active sites have been designed for polymerization reactions, relatively few reports have described a catalytic design in which the catalytic active site does not leave the CD monomer recognition site during the growing step. In a previous design of radical initiators with CDs, the radical-initiating end group leaves the CD monomer recognition site [59–60]. With this molecular design, an included monomer is distant from the radical species and cannot be involved in the direct polymerization. Here, we will observe the effect of monomer recognition of CD on polymerization if a supramolecular polymerization catalyst capable of inserting the monomer between the active and binding sites can be designed. Based on this concept, we have reported that CDs can include and activate lactones to yield a polymer with a single CD at the end of the polymer chain [61–64]. Subsequently, we reported ring-opening metathesis polymerization involving the use of a Ru complex with a CD-derived monophosphine ligand [47]. In the design of the supramolecular polymerization catalysts, monomers are inserted between the initiating end group and the growing polymer chain.

In this study, the monomer recognition site is introduced to a reversible addition–fragmentation chain transfer (RAFT) polymerization system [65–69]. We have synthesized a chain transfer agent (CTA) bearing the CD moiety (CD-CTA) and have investigated this agent’s polymerization behavior. The polymerization rate constant decreased with the addition of competitive molecules, indicating that complexation between CD-CTA and the monomer plays an important role in determining polymerization rate.

## Results and Discussion

### Preparation of α-CD-CTA

**Figure 1:**
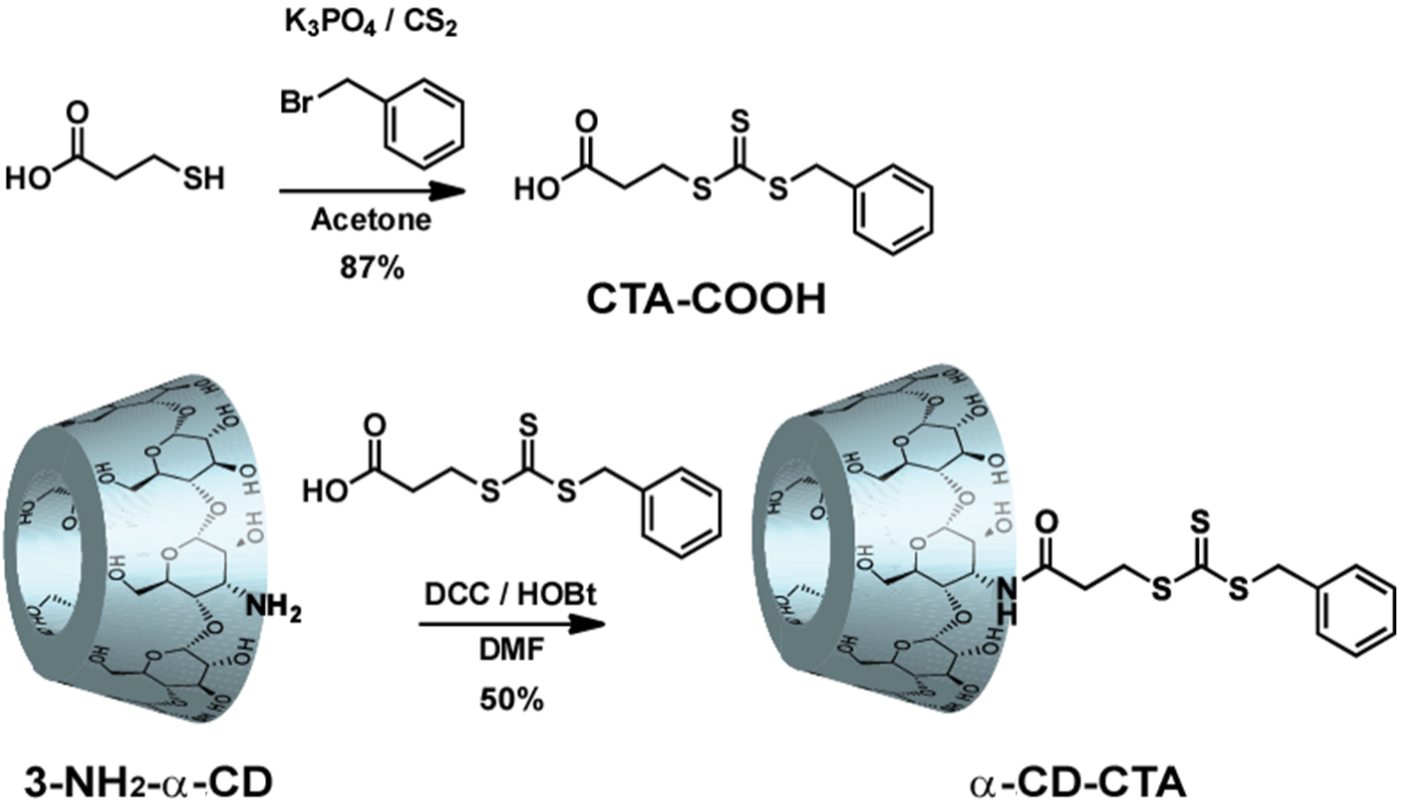
Preparation scheme of α-CD-CTA.

We designed a CTA reagent with α-CD or β-CD. Figure 1 illustrates the preparation of α-CD-CTA. Mercaptopropionic acid was reacted with benzyl bromide, K_3_PO_4_, and carbon bisulfide (CS_2_) in acetone to afford a trithiocarbonyl derivative, a CTA with a carboxylic acid (CTA-COOH). α-CD-CTA was prepared in 50% yield by the reaction of CTA-COOH and 3-NH_2_-α-CD with *N*,*N*'-dicyclohexylcarbodiimide (DCC)/1-hydroxybenzotriazole (HOBt) in DMF. The α-CD-CTA was purified using reverse-phase chromatography. β-CD-CTA was prepared using the same method as α-CD-CTA in 45% yield (see Supporting Information File 1). α-CD-CTA and β-CD-CTA can be dissolved in water. However, the solubility of β-CD-CTA in water was significantly low, leading to the formation of precipitates. β-CD-CTA forms a self-inclusion complex or a supramolecular dimer complex, which was characterized using 2D ROESY NMR (Supporting Information File 1, Figure S4). We focused on the polymerization activity of α-CD-CTA because the β-CD cavity of β-CD-CTA was capped by the CTA unit, inhibiting the molecular recognition property.

### Crystal structure of the α-CD-DMA and β-CD-DMA complexes

**Figure 2:**
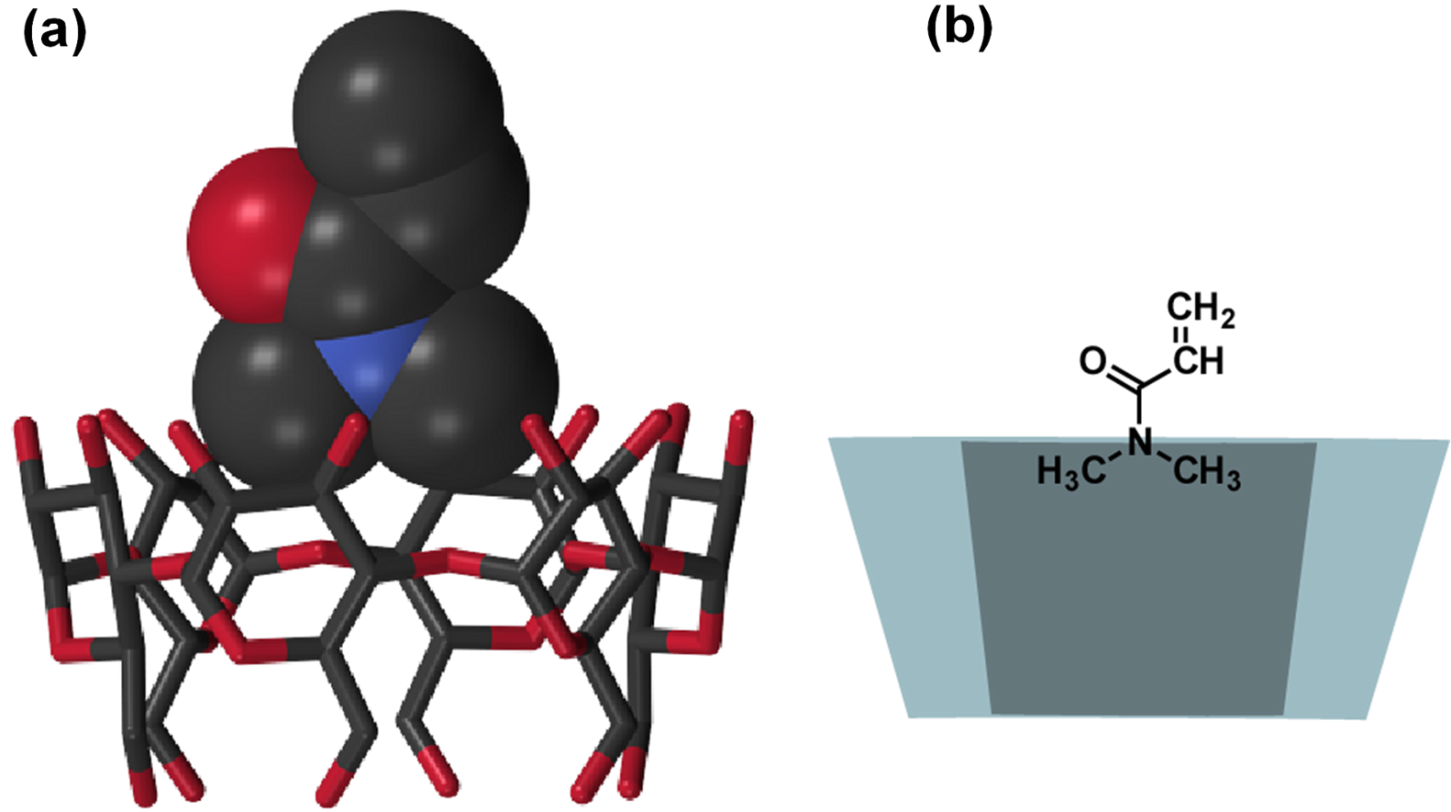
Crystal structure of α-CD with *N*,*N*-dimethylacrylamide (DMA). (a) The structure of an 1:1 inclusion complex between α-CD and DMA. One of the disordered pattern of DMA is shown. DMA molecules outside the α-CD, hydrogen atoms, and water molecules are omitted for clarity. Colors of the atoms are based on CPK coloring. DMA, space-filling model; CD, stick model. (b) A schematic diagram of the inclusion complex of α-CD/DMA.

We chose *N*,*N*-dimethylacrylamide (DMA), acrylic acid (AA), and acrylamide (AAm) as water-soluble vinyl monomers for radical polymerization. Prior to studying the polymerization of vinyl monomers, we investigated the complex formation of CDs with vinyl monomers. When mixing α-CD and DMA, we obtained single crystals suitable for X-ray crystallography analysis. The X-ray crystallography analysis is important to understand the complex in the condensed phase. Figure 2a shows the crystal structure of α-CD with DMA. α-CD formed a head-to-tail channel structure in the crystal. Figure 2b shows the schematic diagram of the crystal structure of α-CD/DMA. The stoichiometry of α-CD and encapsulated DMA was 1:1.

The *N*,*N*-dimethylamino group was located at the wider rim (secondary hydroxy group) of the α-CD cavity and the vinyl group was pointed toward the opposite direction. Based on the crystal structure, it is speculated that the modification of the wider rim of α-CD with the CTA group would bring the vinyl group of DMA and reactive radical species close together during the course of RAFT polymerization.

Figure S5a (Supporting Information File 1) shows the crystal structure of β-CD with DMA. β-CD formed a head-to-head dimeric structure in the crystal. Figure S5b shows a schematic diagram of the crystal structure of β-CD/DMA. The stoichiometry of β-CD and encapsulated DMA was 2:3. For two of the DMA molecules, the *N*,*N*-dimethylamino and the vinyl groups were located at the wider and the narrower rims of the β-CD cavity, respectively. The other one of the DMA molecules was packed between the two β-CD molecules in an equatorial plane.

When mixing α-CD and AA in water, we obtained a powder precipitate of a complex between α-CD and AA, although the crystal structure of α-CD and AA was not solved. The formation of precipitate implied the formation of a host-guest complex between them. A mixture of CDs (α-CD, β-CD and γ-CD) and AAm did not result in a precipitate, indicating that the affinities of the CDs for AAm were low.

### Polymerization of vinyl monomers mediated by α-CD-CTA

α-CD-CTA-mediated polymerizations of water-soluble vinyl monomers were performed in aqueous media, where the strong molecular recognition property of α-CD is expected due to the hydrophobic effect. As a water-soluble radical initiator, we selected 2,2'-azobis[2-(2-imidazolin-2-yl)propane] dihydrochloride (VA-044). Two hundred equivalents (equiv) of monomers, 1 equiv of α-CD-CTA, and 0.4 equiv of VA-044 were mixed in water (monomer: 1 mol/kg). After the reactants were mixed and degassed, the sample tube was heated at 45 °C in an Ar atmosphere. After 24 hours, the polymer solution was lyophilized. The resulting polymers were dissolved in 8 mL MeOH or chloroform, then reprecipitated into 80 mL diethyl ether. This cycle repeated twice to remove monomer. The structures of the obtained polymers were characterized by ^1^H NMR spectroscopy (Figure S6, Supporting Information File 1). The ^1^H NMR signals of the benzyl group and those of the CD moiety of α-CD-CTA were both observed in the obtained polymer, which indicates that both groups did not leave the polymer main chain after the radical polymerization and that α-CD-CTA functioned as a good chain transfer agent. Some end groups are formed from the VA 044 initiator.

Table 1 shows results for VA-044-initiated polymerizations of DMA, AA and AAm. Polymers’ molecular weights and distributions were determined by gel permeation chromatography (GPC). The polymerization of DMA and AA mediated by α-CD-CTA gave polymers with similar molecular weights (12.8 and 14.8 kDa) and narrow distributions in good yields (85–88%) (Table 1, entries 2 and 5). In contrast, in the absence of α-CD-CTA, free radical polymerization with VA-044 gave polymers with higher molecular weights and wider distributions (Table 1, entries 1 and 4). In order to investigate the effect of encapsulation of monomer by α-CD moiety, the reactions in the presence of 1,6-hexanediol (C_6_ diol) as a competitive guest molecule were also investigated. C_6_ diol was selected as a non-ionic molecule with a high association constant to α-CD (*K*_a_ = 134 M^−1^ ) [70]. In the presence of C_6_ diol, the reaction yields dropped to 63–72%, whereas the molecular weights of the polymers increased (22.9 and 19.4 kDa in entries 3 and 6, respectively). It is considered that by inhibiting the molecular recognition of α-CD, the inclusion complexation ratio between the monomer and α-CD-CTA was decreased, which lead to lower yields and higher molecular weight of the resulting polymers due to preceding free radical polymerization. In the reaction of AAm monomer, which has a low affinity with α-CD, the properties of the obtained polymers were similar regardless of the presence of the α-CD-CTA or the competitive C_6_ diol guest. The polymerization of AAm mediated by α-CD-CTA gave polymers with slightly narrower distributions. From the series of experiments, it was demonstrated that the molecular recognition property of α-CD-CTA effectively affected the chain transfer step.

**Table 1:** Polymerizations of water-soluble vinyl monomers, mediated by α-CD-CTA^a^.

Entry	CTA^b^	Monomer^b^	Competitor^c^	*M*_n_ / 10^3 d^	*M*_w _*/ M*_n_^d^	Yield%

1	–	DMA	–	9679	>10	96
2	α-CD-CTA	DMA	–	**12.8**	**1.2**	**85**
3	α-CD-CTA	DMA	C_6_ diol	22.9	1.3	63
4	–	AA	–	45.9	>10	98
5	α-CD-CTA	AA	–	**14.8**	**1.2**	**88**
6	α-CD-CTA	AA	C_6_ diol	19.4	1.2	72
7^e^	–	AAm	–	3841	>10	98
8^e^	α-CD-CTA	AAm	–	**10.4**	**1.3**	**98**
9^e^	α-CD-CTA	AAm	C_6_ diol	9.9	1.4	99

^a^Polymerization was performed at 45 °C for 24 h, with VA-044 as an initiator. ^b^[Monomer]_0_/[CTA]_0_/[I]_0_ = 200/1/0.4 ([Monomer]_0_ = 1 M). ^c^[Competitor]_0_/[CTA]_0_ = 50/1. ^d^*M*_n_ and *M*_w_/*M*_n_ were determined by GPC using polystyrene sulfonate sodium salt (PSSNa) or polyacrylamide (PAAm) as references. ^e^Polymerization of AAm was performed in acetate buffer [71–72].

### Rate and mechanism of α-CD-CTA-mediated polymerization

We investigated the polymerization rate of DMA mediated by α-CD-CTA and the effect of the competitive molecule in water. A mixture of DMA (200 equiv), α-CD-CTA (1 equiv), and VA-044 (0.4 equiv) in D_2_O (0.25 mol/kg) was degassed and heated at 45 °C in an Ar atmosphere. When a concentration of 1 mol/kg was used, the polymerization rate was too fast to trace during the initial stages of polymerization. Therefore, we chose to utilize a concentration of 0.25 mol/kg because this concentration allowed the polymerization rate to be easily followed. At regular time intervals, a 0.6 mL sample was collected and analyzed using ^1^H NMR spectroscopy. The conversions were calculated using the ratio between the integral values for the polymer main chain and the vinyl group of the DMA monomer.

**Figure 3:**
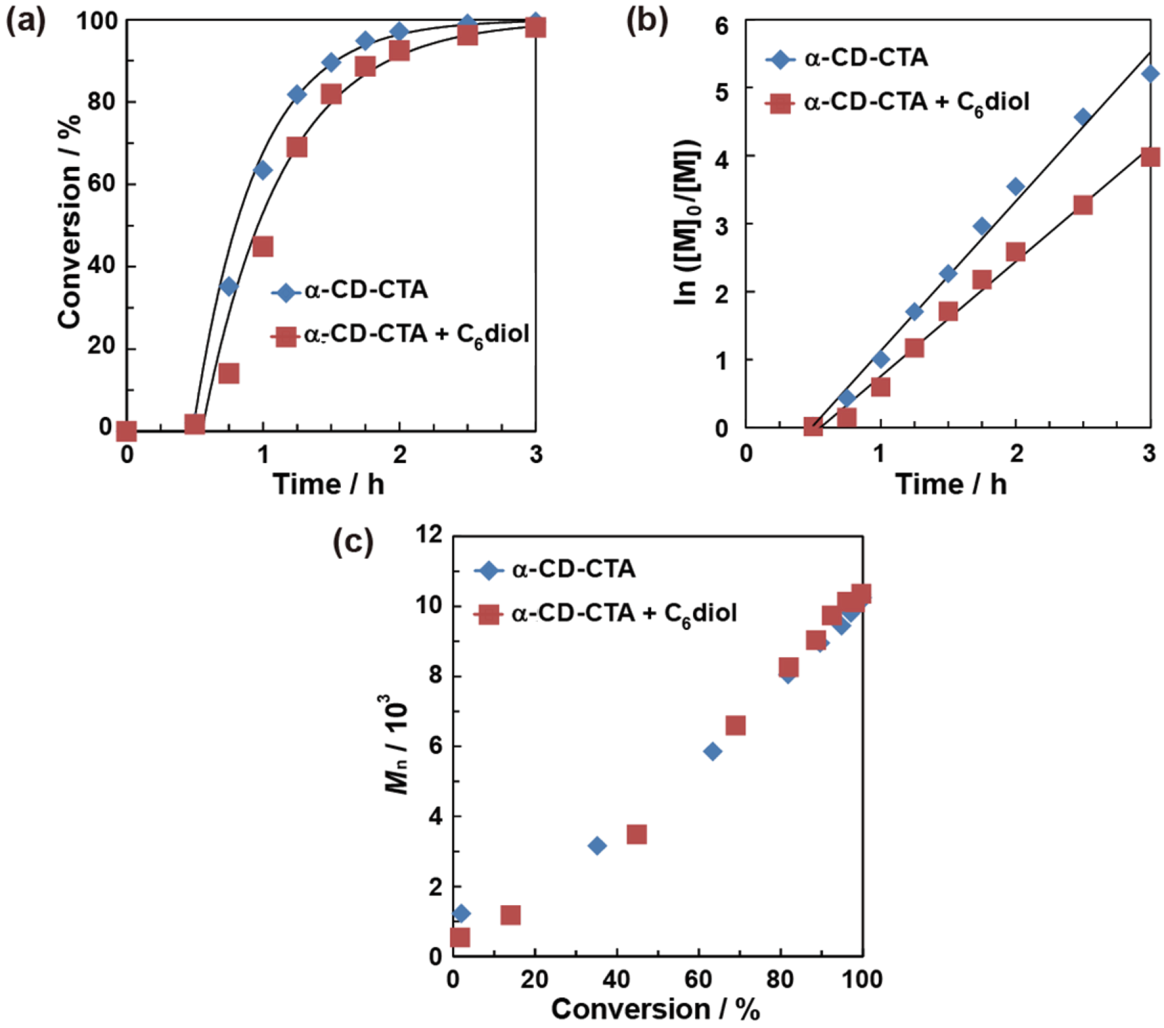
Time-conversion curves (a), kinetic plots (b) and plots of number-average molecular weight (*M*_n_) versus conversion (c) for the α-CD-CTA-mediated polymerization of DMA. The blue rhombic plots correspond to the α-CD-CTA-mediated polymerization of DMA. The red square plots correspond to the α-CD-CTA/C_6_ diol-mediated polymerization of DMA.

Figure 3a, b and c shows time-conversion curves, kinetic plots, and *M*_n_-conversion for the polymerization of DMA mediated by α-CD-CTA. The time-conversion plots indicate the induction period in the presence and absence of C_6_ diol. Kinetic rates were determined using the least-square method after the induction period. The presence of C_6_ diol made a clear difference in the polymerization rate by α-CD-CTA. The kinetic rate was faster for α-CD-CTA (*k* = 2.2 h^−1^) than for α-CD-CTA/C_6_ diol (*k* = 1.7 h^−1^). This finding supports that C_6_ diol inhibits monomer recognition by the α-CD cavity, which was also indicated by the series of experiments in Table 1.

**Figure 4:**
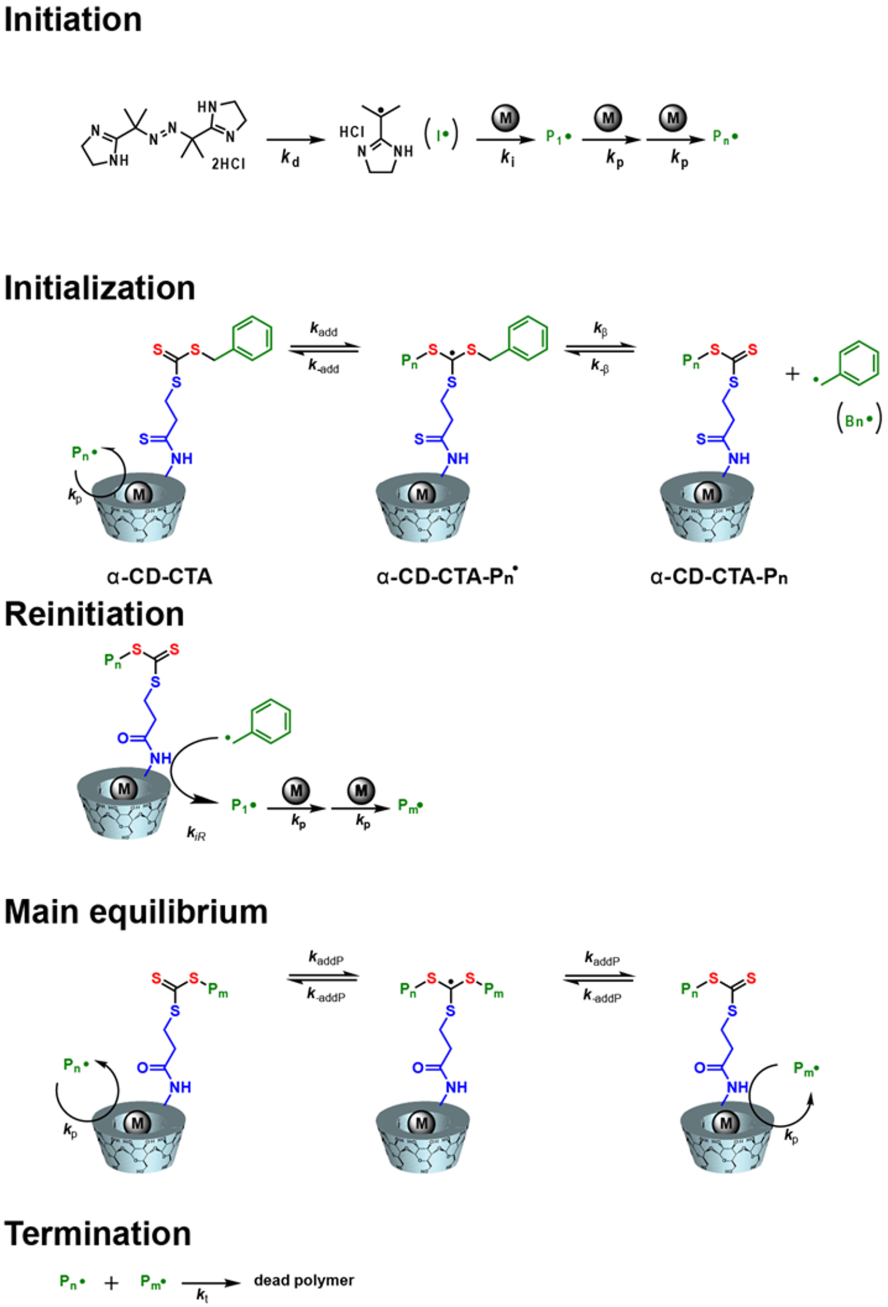
Proposed polymerization mechanism for a water-soluble vinyl monomer with α-CD-CTA as a chain transfer reagent. 2,2'-Azobis[2-(2-imidazolin-2-yl)propane] dihydrochloride (VA-044) was used as a water-soluble radical initiator.

Figure 4 shows the proposed polymerization mechanism of a water-soluble vinyl monomer with α-CD-CTA. The radical (I**^•^**) derived from VA-044 attacks the monomer to produce a polymeric radical (P*_n_***^•^**). P*_n_***^•^** reacts with α-CD-CTA to generate the intermediate α-CD-CTA-P*_n_***^•^**. α-CD-CTA-P*_n_***^•^** is held in an equilibrium state with the macro-CTA, α-CD-CTA-P*_n_*, and the benzyl radical (Bn**^•^**) with re-initiation activity. As demonstrated by the inhibition experiments, the yields and molecular weights of the polymers were affected by the molecular recognition property of α-CD-CTA. Based on these results, P*_n_***^•^** and Bn**^•^** preferentially attack the included monomer to afford the growing polymer in high yield. Therefore, the kinetic rate is faster for α-CD-CTA than for α-CD-CTA/C_6_ diol, which produced polymers with narrow distribution. In the polymerization reaction, some of the polymer chains (P*_n_*•) form dead chains through termination or irreversible chain transfer. However, the *M*_w_/*M*_n_ of the polymer mediated by α-CD-CTA agents that the termination reaction is suppressed by α-CD-CTA compared to free radical polymerization.

## Conclusion

We studied the radical polymerization of water-soluble vinyl monomers using CD-CTA with molecular recognition property. α-CD was found to include a DMA monomer in a 1:1 manner, which was characterized using single X-ray crystallography analysis. The polymerization of DMA with α-CD-CTA resulted in poly(DMA) with a narrow distribution in high yield, whereas a low yield was obtained for polymerization in the presence of C_6_ diol, a competitive guest molecule for α-CD. In contrast, the polymerization of AAm, which had a lower affinity for α-CD, was not affected by α-CD-CTA in either the presence or absence of C_6_ diol. These results indicate that the chain transfer reagent modified with a host molecule provided the site for a reaction between the end group of the growing polymer and monomers. Currently, we are investigating the preparation of supramolecular catalysts with a chain transfer reagent with two CDs to recognize monomers and the growing polymer chain, and exploring its function mimicking a biological molecular clamp.

## Supporting Information

File 1 The preparation of α-CD-CTA and β-CD-CTA and typical polymerization methods.

File 2 Crystallographic information file for α-CD-DMA.

File 3 Crystallographic information file for β-CD-DMA.
