# Supplementary material for: Radical polymerization by a supramolecular catalyst: cyclodextrin with a RAFT reagent
Source: Beilstein J Org Chem. 2016 Nov 22;12:2495–502. doi: 10.3762/bjoc.12.244 (PMC5238571; doi:10.3762/bjoc.12.244)
Supplement: File 1 — The preparation of α-CD-CTA and β-CD-CTA and typical polymerization methods. [file Beilstein_J_Org_Chem-12-2495-s001.pdf]

Supporting Information

for

Radical polymerization by a supramolecular catalyst: cyclodextrin with a RAFT reagent

Kohei Koyanagi[1], Yoshinori Takashima[1], Takashi Nakamura[§,1], Hiroyasu Yamaguchi[1] and Akira Harada*[1,2]

Address: [1]Department of Macromolecular Science, Graduate School of Science, Osaka University, Toyonaka, Osaka 560-0043, Japan and [2]JST-ImPACT, Chiyoda-ku, Tokyo 100-8914, Japan

[§]Current affiliation: Faculty of Pure and Applied Sciences, University of Tsukuba.

Email: Akira Harada - harada@chem.sci.osaka-u.ac.jp

*Corresponding author

The preparation of α-CD-CTA and β-CD-CTA and typical polymerization methods

Experimental section

Materials

K_3PO_4, 2,2'-azobis[2-(2-imidazolin-2-yl)propane]dihydrochloride (VA-044), and acrylamide were purchased from WAKO Pure Chemical Industries, Ltd. α-Cyclodextrin (α-CD) was purchased from Junsei Chemical Co., Ltd., and recrystallized from water prior to use. CS_2, 3A-amino-3A-deoxy-(2AS,3AS)-α-CD hydrate (3-NH_2-α-CD) and 3A-Amino-3A-deoxy-(2AS,3AS)-β-CD hydrate (3-NH_2-β-CD) were purchased from Tokyo Chemical Industry Co., Ltd. *N,N*'-Dicyclohexylcarbodiimide (DCC), 1-hydroxybenzotriazole (HOBt), NaOH, acetic acid, and sodium acetate were purchased from Nakalai Tesque, Inc. 3-Mercapto propionic acid was purchased from Sigma-Aldrich Co. Acetone was dried with 3Å molecular sieves overnight prior to use. Dry solvents were purified by passage through solvent purification columns containing activated alumina. D_2O was purchased from Cambridge Isotope Laboratories, Inc. and distilled prior to use in time-conversion experiments.

All chemicals except monomers and α-CD were used as supplied. Monomers were purified to remove inhibitors prior to use. Acrylamide (AAm) was recrystallized from water. Acrylic acid (AA) and *N,N*'-dimethylacrylamide (DMA) were purified by distillation at reduced pressure.

Measurements and procedures.

The ^{1}H NMR and ^{13}C NMR spectra were recorded at 600 MHz with a VNS 600 NMR spectrometer, 500 MHz with a JEOL JNM-ECA 500 NMR spectrometer and at 300 MHz with a Varian Mercury 300 NMR spectrometer. Positive-ion matrix-assisted laser desorption ionization time of flight (MALDI-TOF) mass spectrometry experiments were performed on a BRUKER autoflex™ speed MALDI-TOF system calibrated by dihydoxybenzoic acid (DHBA) and peptide standard (Aldrich).

Gel permeation chromatography (GPC) was performed in 10 mmol LiBr in formamide (0.30 mL/min, 40 °C) using an TOSOH HLC-8320GPC EcoSEC® equipped with a TOSOH TSK gel α-M column to determine molecular weights. M_n and M_n/M_w of the obtained polymers were measured with respect to polystyrene sulfonate sodium salt (PSSNa) standards (American polymer standards Corp., Mentor, Ohio), and polyacrylamide (PAAm) standards (American polymer standards Corp., Mentor, Ohio).

The crude products were purified by reversed-phase preparative HPLC (Waters 2487, colomn: nakalai tesque COSMOSIL 5C18-MS-II Prep column, solvent: water/acetonitrile, flow rate 16 mL/min)

Single crystal X-ray crystallographic measurements

Single crystal X-ray crystallographic measurements were performed using a Rigaku RAXIS-RAPID imaging plate diffractometer with MoKα radiation. Obtained data were calculated using the Crystal Structure 4.0.1. crystallographic software package except for refinement, which was performed using SHELXL-97. CCDC 1500212 and 1500213 contain the data for the structures. The data can be obtained free of charge from The Cambridge Crystallographic Data Centre via www.ccdc.cam.ac.uk/getstructures.

Preparation of single crystals of α-CD-*N,N'*-dimethyl acrylamide complex

To 5 mL of an aqueous α-CD solution (75 mM) in a sample tube, *N,N'*-dimethyl acrylamide (5 mmol) was added and the mixture was shaken until a clear solution was obtained. The mixture was left standing for days and the obtained crystals were analyzed by X-ray diffraction.

Aqueous polymerization of acrylamide (AAm)

α-CD-CTA (0.01 mmol), acrylamide (144 mg, 2 mmol), VA-044 (0.13 mg, 4 μmol) were dissolved in acetate buffer (4 mL). After three successive freeze-and-thaw cycles, the Schlenk flasks were subsequently immersed in an oil bath at 45 °C, and the polymerization was allowed to proceed for 24 h before being quenched with by immersion in MeOH/dry ice bath. The resulting solution was purified by reprecipitated in MeOH three times and dried in vacuo. The resulting polymer was characterized by ^{1}H-NMR and GPC (respect to PAAm standard).

Aqueous polymerization of Acrylic acid (AA)

α-CD-CTA (0.01 mmol) and VA-044 (1.30 mg, 4 μmol) were dissolved in freshly distilled water (2 mL) under Ar atmosphere. After adding acrylic acid (140 μL, 2 mmol), the solution was degassed by two successive freeze-and-thaw cycles. The schlenk flasks were subsequently immersed in an oil bath at 45 °C, and the polymerization was allowed to proceed for 24 h before being quenched by immersion in an MeOH/dry ice bath. The resulting solution was freeze-dried to obtain the crude polymer. The crude polymer was dissolved in MeOH (8 mL), then reprecipitated in diethyl ether (80 mL) for three times and dried in vacuo. The resulting polymer was characterized by ^{1}H NMR

and GPC (respect to PSSNa standard).

Aquaous polymerization of *N*,*N'*-dimethylacrylamide (DMA)

The polymerization of DMA was carried out in an analogous manner to that of acrylic acid using DMA (205 μL, 2 mmol). After freeze-drying the reaction mixture, the crude polymer was dissolved in DCM (8 mL), then reprecipitated in diethyl ether (80 mL) three times and dried in vacuo. The resulting polymer was characterized by ^{1}H NMR and GPC (respect to PAAm standard).

Time-conversion measurement

The entire solution was diluted 4 times to slow down the polymerization rate ([Monomer] = 0.25 M, [CTA] = 1.25 mM, [I] = 0.5 mM).

α-CD-CTA (10 μmol) and VA-044 (1.3 mg, 4 μmol) were dissolved in freshly distilled D_2O (8 mL) under Ar atmosphere. After adding DMA (200 μL, 2 mmol), the solution was degassed by two successive freeze-and-thaw cycles. The Schlenk flasks equipped with 3 way valves were subsequently immersed in an oil bath at 45 °C. 600 μL of reaction mixture was removed after 0.5, 1, 1.5, 2.0, 3.0, 4.0 and 6.0 h, diluted with D_2O and analyzed by ^{1}H NMR and GPC. The conversion was determined by comparing the peak area corresponding to vinyl unit (CH_2=C*H*-) and polymer main chain (-CH_2-C*H*<).

Preparation

Preparation of 3-(benzylthiocarbonothioylthio)propanoic acid (CTA-COOH).

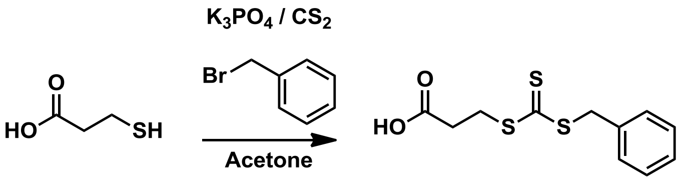

CTA-COOH was synthesized in a similar manner as described in [1]. To a stirred suspension of K_3PO_4 (4.0 g, 18.8 mmol) in acetone (10 mL), 3-mercaptopropionic acid (821 μL, 9.43 mmol) was added and stirring for 10 min. CS_2 (1.7 mL, 28.3 mmol) was added and the solution turned bright yellow. After stirring for 20 min, benzyl bromide (1.13 mL, 9.43 mmol) was added and an instant precipitation of KBr was noted. After stirring for 30 min, the solvent was removed under reduced pressure and the residue was added to brine (100 mL) and extracted with CH_2Cl_2 (2 × 125 mL) washed with brine (2 × 50 mL). After drying of the organic extracts over $MgSO_{4,}$ the solvent was removed under reduced pressure. The residue was dissolved in ethyl acetate and precipitated in hexane to furnish CTA-COOH as yellow powder. Yield: 2.22 g (87%).

^{1}H NMR (500 MHz, $CDCl_3$, 25 °C): δ = 7.38 (2H, t, Ph (m-)), 7.34 (2H, ttt, Ph (o-)), 7.29 (1H, ttt, Ph (p-)), 4.68 (2H, s, -S(CS)S-CH_2-Ph), 3.54 (2H, t, J = 6.9 Hz, -CH_2-CH_2-S(CS)S-), 2.68 (2H, t, J = 6.9 Hz, HOOC-CH_2-CH_2-).

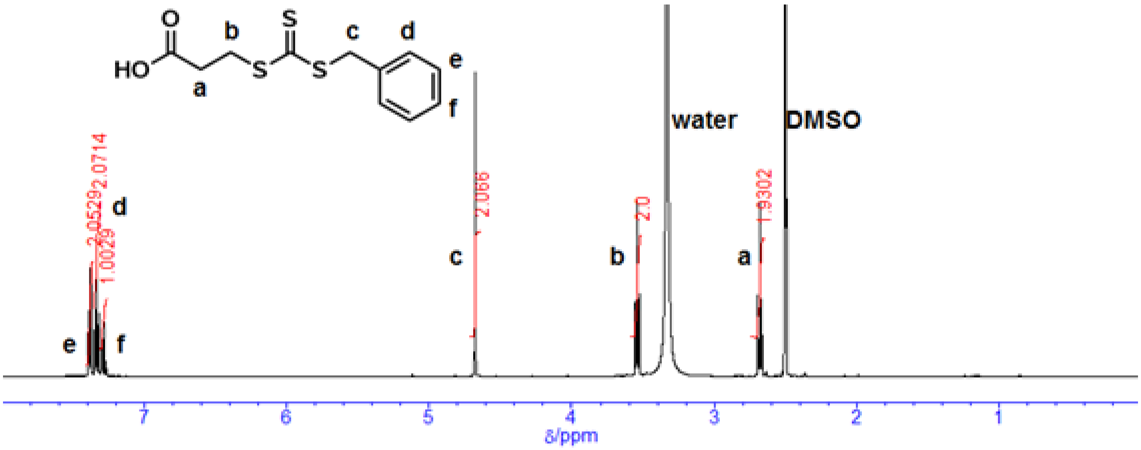

Figure S1: ^{1}H NMR spectrum of CTA-COOH (500 MHz, DMSO-d_6, 25 °C)

Table S1: The HPLC gradient program for the preparation of α-CD-CTA and β-CD-CTA

Time / min.	H_2O / %	MeCN / %	curve program
0	100	0	6(Linear)
40	60	40	6(Linear)
60	100	0	6(Linear)

The flow rate was 16 mL/min.

Preparation of α-CD-CTA.

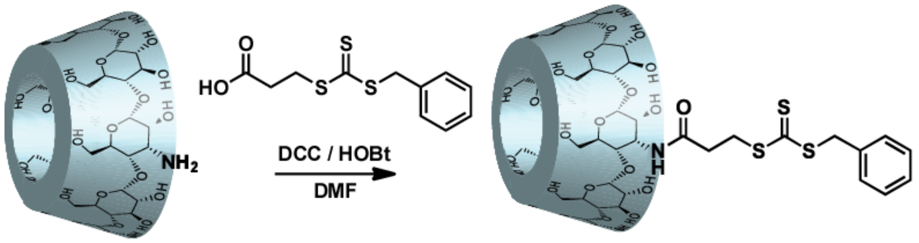

To a solution of 3-NH_2-α-CD (400 mg, 0.41 mmol) in dry DMF (5 mL) in an ice bath, mixture of CTA-COOH (145 mg, 0.53 mmol), DCC (111 mg, 0.54 mmol), and HOBt (73 mg, 0.54 mmol) in DMF (3 mL) was added and stirred 6 h at room temperature then 2 h at 50 °C. The reaction mixture was centrifuged and the supernatant was reprecipitated in acetone (40 mL). The crude product was purified by (on a DIAION HP-20 column) reversed phase HPLC(water 100% to acetonitrile 40 %). Yield: 252 mg (50%)

^{1}H NMR (500 MHz, $CDCl_3$, 25 °C): δ= 7.81 (d, J = 8.9 Hz, 1H, -CON*H*-), 7.38 (2H, t, Ph (*m*-)), 7.34 (2H, ttt, Ph (*o*-)), 7.29 (1H, ttt, Ph (*p*-)), 5.88-5.16 (m, 11H, O2*H* and O3*H*), 4.87-4.78 (m, 6H, C1*H*), 4.70-4.44 (m, 8H, O6*H* and C*H*$_2$-Ph), 4.02-3.51 (m, 26H, C3*H*, C5*H*, and C6*H* and -CH_2-C*H*$_2$-S(CS)S-), 3.44-3.12 (12H, m, overlaps with HOD, C2*H* and C4*H*), 2.54 (2H, t, HOOC-C*H*$_2$-CH_2-).

Positive ion MALDI-TOF Mass. Calcd. for $C_{47}H_{71}NO_{30}S_3Na$ ([M +Na]+): 1248.31; Found: 1248.2.

Elemental analysis calcd. for $C_{47}H_{71}NO_{30}S_3 \cdot 7H_2O$: C, 41.74; H, 6.34; N, 1.04. Found: C, 41.82; H, 6.05; N, 1.11 (error 0.29%)

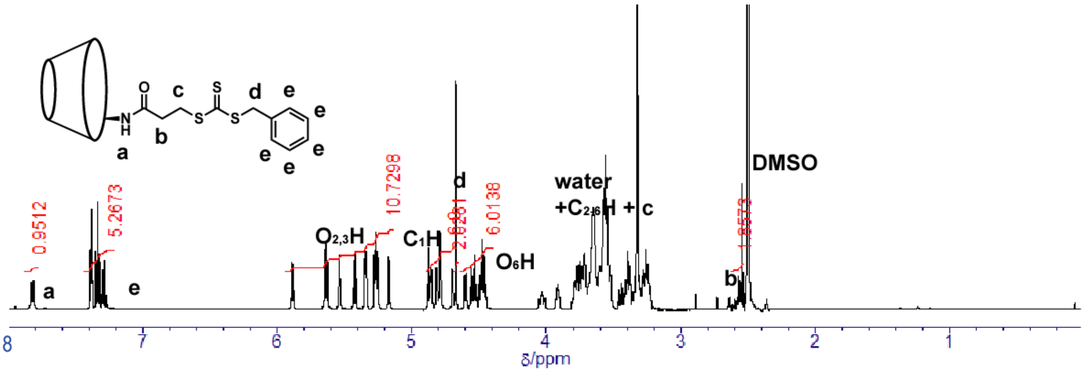

Figure S2: ^{1}H NMR spectrum of α-CD-CTA (500 MHz, DMSO-d_6, 25 °C)

Preparation of β-CD-CTA.

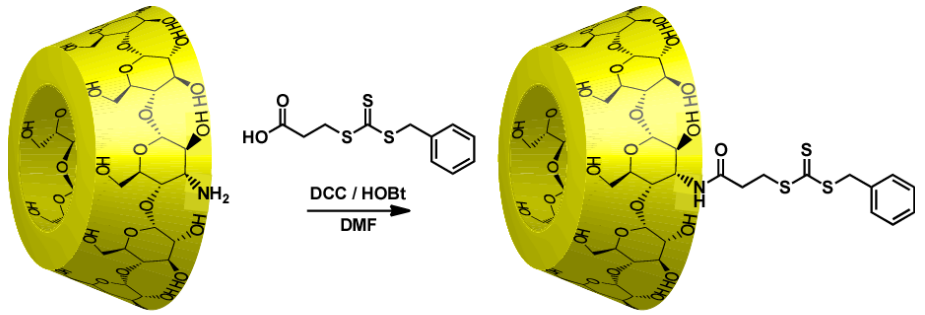

β-CD-CTA was prepared in an analogous manner to that of α-CD-CTA using 3-NH_2-β-CD (400 mg, 0.35 mmol). Yield: 220 mg (45%)

^{1}H NMR (500 MHz, DMSO-d_6, 25 °C): δ=7.81 (d, *J* = 8.9 Hz, 1H, -CON*H*-), 7.37 (2H, t, Ph (*m*-)), 7.32 (2H, ttt, Ph (*o*-)), 7.28 (1H, ttt, Ph (*p*-)), 5.88-5.16 (m, 13H, O2*H* and O3*H*), 4.88-4.80 (m, 7H, C1*H*), 4.71-4.39 (m, 9H, O6*H* and C*H*$_2$-Ph), 4.02-3.51 (m, 30H, C3*H*, C5*H*, and C6*H* and -CH_2-C*H*$_2$-S(CS)S-), 3.44-3.12 (14H, m, overlaps with HOD, C2*H* and C4*H*), 2.55 (2H, t, HOOC-C*H*$_2$-CH_2-).

Positive ion MALDI-TOF Mass. Calcd. for $C_{53}H_{81}NO_{35}S_3Na$ ([M +Na]+): 1248.31; Found: 1248.2.

Elemental analysis calcd. for $C_{53}H_{81}NO_{35}S_3 \cdot 8H_2O$: C, 41.54; H, 6.38; N, 0.91. Found: C, 41.49; H, 6.12; N, 0.88 (error 0.26%)

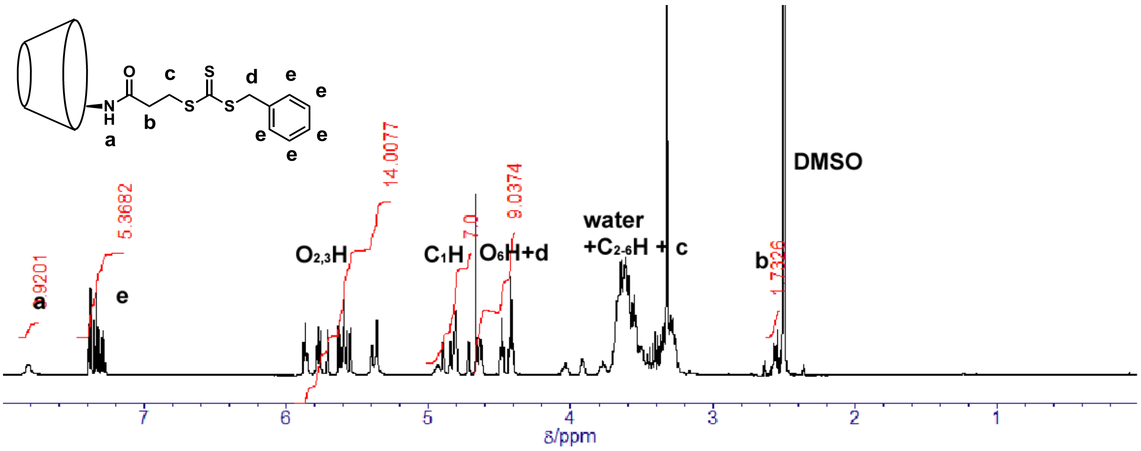

Figure S3: ^{1}H NMR spectrum of β-CD-CTA (500 MHz, DMSO-d_6, 25 °C)

2D ROESY of β-CD-CTA

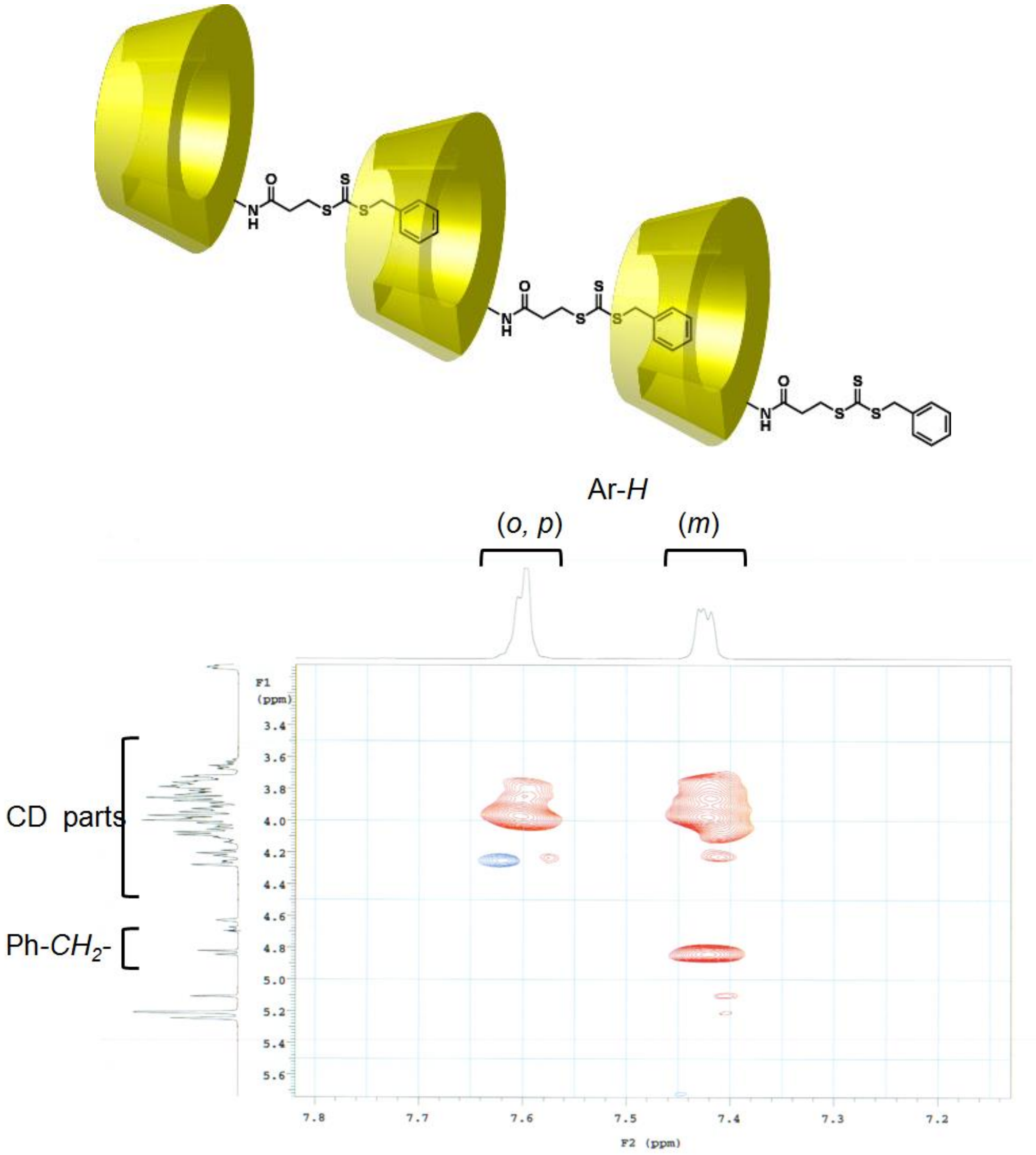

Figure S4: 2D ROESY spectrum of β-CD-CTA in D_2O.

Crystal structure of the β-CD/DMA complex

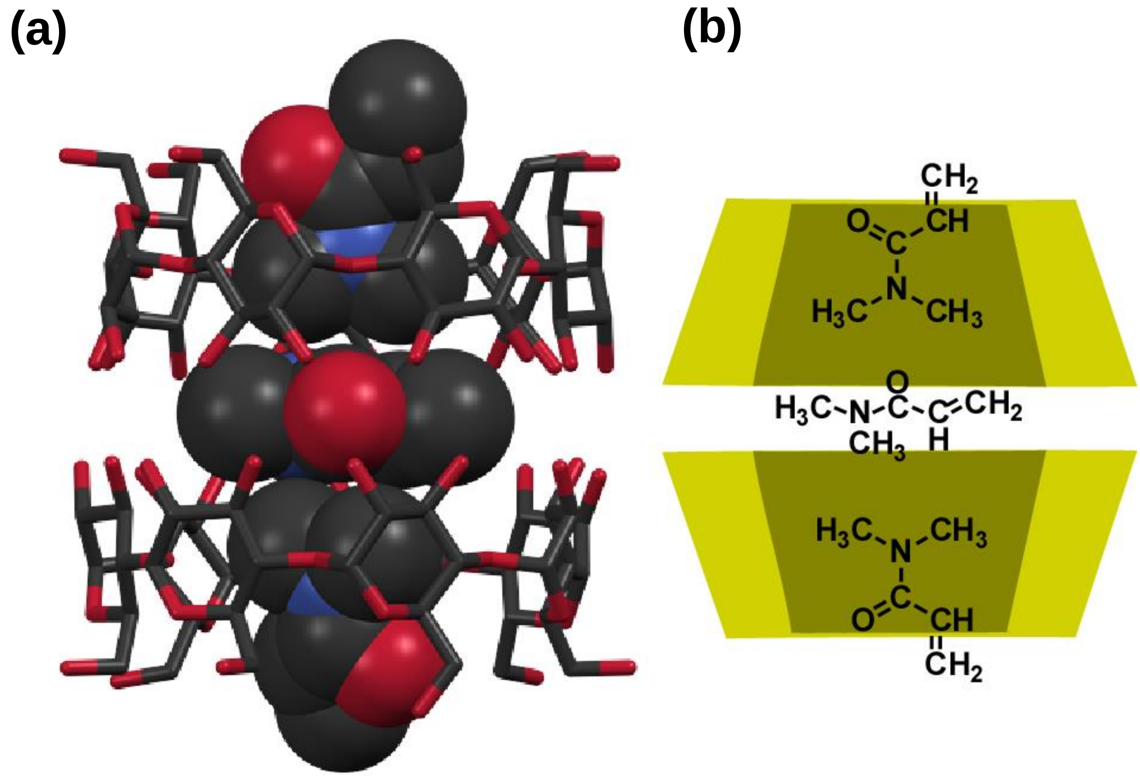

Figure S5: Crystal structure of β-CD with *N*,*N*'-dimethylacrylamide (DMA). (a) The structure of a 2:3 inclusion complex between β-CD and DMA. DMA molecules outside the β-CD, hydrogen atoms, and water molecules are omitted for clarity. Colors of the atoms are based on CPK coloring. DMA, space-filling model; CD, stick model. (b) A schematic diagram of the inclusion complex of β-CD/DMA

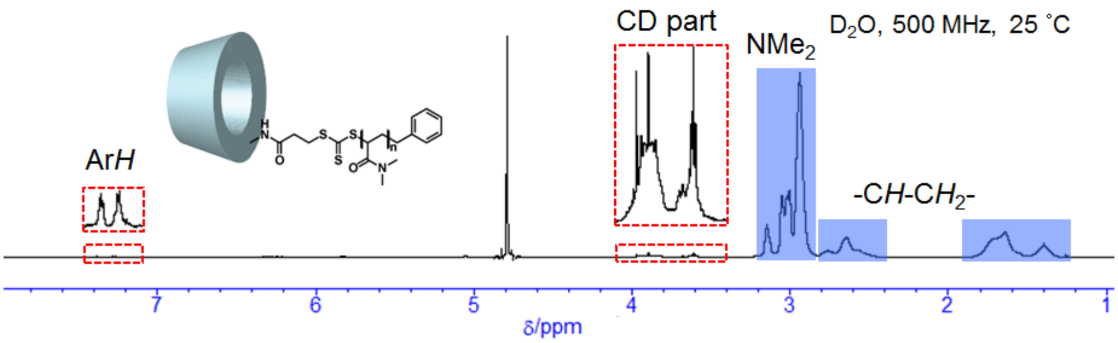

Figure S6: ^{1}H NMR spectrum of poly(DMA) mediated with α-CD-CTA (500 MHz, D_2O-d_6, 25 °C)

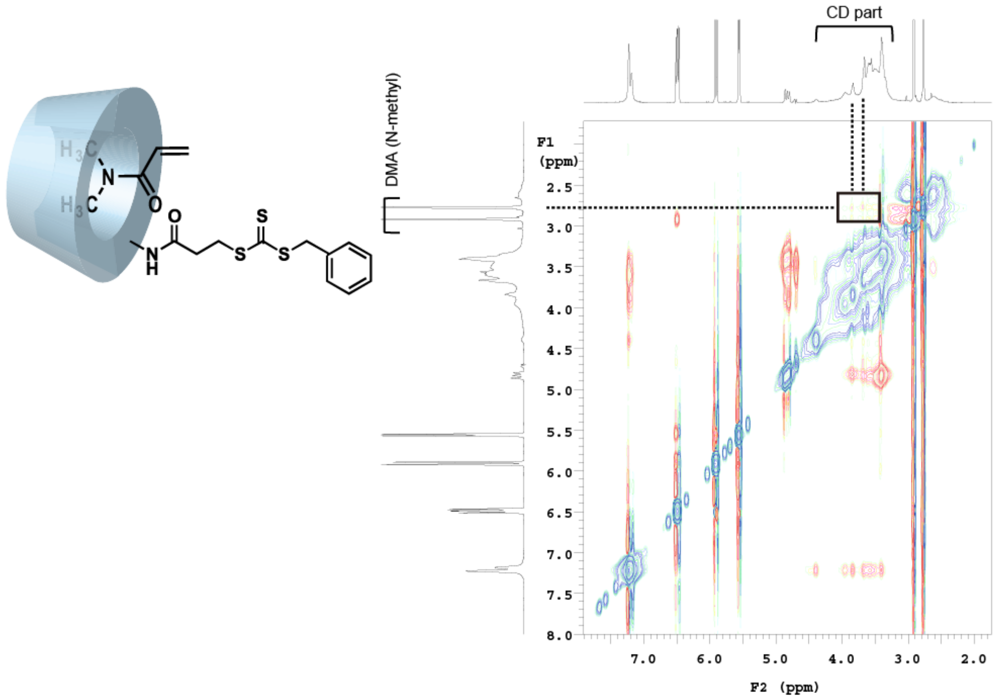

Figure S7: 2D ROESY spectrum of α-CD-CTA with DMA in D_2O

Refference

1. Skey, J.; O'Reilly, R. K. *Chem. Commun.* **2008,** 35, 4183–4185.
